# Biologically effective doses for α/β = 10 (BED_10_) of <80 Gy versus ≥80 Gy in stereotactic body radiotherapy for pancreatic cancer: a retrospective single-center analysis

**DOI:** 10.3389/fonc.2026.1850091

**Published:** 2026-08-26

**Authors:** Qian Wang, Shaoyu Cao, Zheng Zheng, Changwen Bo, Na Li, Qiuning Li, Yao Fan

**Affiliations:** 1 Department of Oncology, The First Hospital of Hebei Medical University, Shijiazhuang, China; 2 Department of Oncology and Radiotherapy, The First People’s Hospital of Pingdingshan, Pingdingshan, China; 3 Hebei Medical University, Shijiazhuang, China

**Keywords:** clinical efficacy, overall survival, pancreatic cancer, progression-free survival, stereotactic body radiation therapy

## Abstract

**Background:**

The optimal biologically effective dose (BED_10_) for stereotactic body radiation therapy (SBRT) in pancreatic cancer remains debated. This study evaluated whether BED_10_ ≥80 Gy improves outcomes compared with <80 Gy in a heterogeneous patient population.

**Methods:**

46 patients with pancreatic cancer treated with SBRT (May 2021–December 2023) were retrospectively analyzed. Overall survival (OS), progression-free survival (PFS), and local control (LC) were estimated using the Kaplan–Meier method with log-rank testing. Multivariable Cox regression was performed adjusting for disease stage, chemotherapy, tumor size, dose group, and fiducial marker status, with exploratory interaction and subgroup analyses.

**Results:**

Among the 46 patients (10 Stage I/II, 17 locally advanced, 19 metastatic), median OS was 13.0 months (95% CI: 9.9–16.1), with 1-year OS of 51.2% (<80 Gy) vs. 53.3% (≥80 Gy) (log-rank p=0.442). Multivariable analysis showed no significant dose effect (HR 1.472, p=0.342). Independent prognostic factors were disease stage (HR 0.274, p=0.006), tumor size (HR 0.399, p=0.026), chemotherapy (HR 0.410, p=0.029), and absence of fiducial markers (HR 4.224, p=0.006). No significant dose × stage (p=0.373) or dose × chemotherapy (p=0.104) interactions were observed. In 27 patients with evaluable imaging, LC was 92.6% and median PFS was 8.0 vs. 10.0 months (log-rank p=0.605).

**Conclusion:**

In this single-center retrospective analysis, BED_10_ ≥80 Gy was not associated with a statistically significant survival benefit compared with <80 Gy. Independent prognostic factors included disease stage, tumor size, and chemotherapy. The favorable local control and absence of grade ≥3 toxicity suggest that CyberKnife-based SBRT is effective and safe across the studied dose range. However, the heterogeneous study population (41% metastatic) and narrow dose distribution limit definitive conclusions regarding dose-response. Larger prospective studies with homogeneous cohorts are needed to determine whether dose escalation beyond 80 Gy confers meaningful clinical benefit, particularly in patients with locally advanced disease.

## Introduction

Pancreatic cancer (PC), of which pancreatic ductal adenocarcinoma (PDAC) represents the most common histology, remains one of the most lethal malignancies, with a mortality-to-incidence ratio approaching 94% and a 5-year survival rate of approximately 12% (1, 2). Surgical resection offers the only potential for cure; however, fewer than 20% of patients present with resectable disease at diagnosis (3). Even among patients undergoing curative-intent resection, prognosis remains poor due to high rates of both systemic metastasis and local recurrence, with regional recurrence rates reaching 85% following resection alone (4). Thus, effective local-regional control remains a critical challenge.

For patients with unresectable disease or high risk of recurrence, multimodal approaches integrating chemotherapy with radiotherapy (RT) are essential (5). Historically, conventional fractionated radiotherapy (CFRT) delivered daily over 5–6 weeks was the standard of care. However, Stereotactic Body Radiotherapy (SBRT) has emerged as a widely adopted treatment option, characterized by high-precision dose delivery over 1–5 fractions with sharp dose fall-off, enabling ablative doses to the tumor while minimizing dose to adjacent organs at risk (OARs) (6–8). The optimal radiation fractionation strategy—whether conventionally fractionated radiotherapy, moderate hypofractionation, or SBRT—remains an area of active investigation, and the balance among local control, toxicity, and overall survival continues to be refined.

The scientific rationale for dose escalation in pancreatic SBRT is supported by a growing body of evidence. The landmark MD Anderson experience reported by Krishnan et al. demonstrated that focal radiation dose escalation (BED_10_ ≥70 Gy) was the only modifiable factor associated with improved OS in patients with locally advanced pancreatic cancer (LAPC) receiving induction chemotherapy and consolidative chemoradiation (9). More recently, the PAULA-1 experience by Arcelli et al. explored ablative dose levels and reported favorable LC with ablative SBRT in selected patients (10, 11). More recently, a prospective phase 1 dose-escalation trial from Memorial Sloan Kettering Cancer Center and the University of Colorado confirmed the safety of 3-fraction SBRT dose escalation up to 50 Gy (BED_10_ = 133 Gy) in LAPC (12). Despite this evidence, achieving consistently ablative doses (BED_10_ >100 Gy) in pancreatic SBRT is often technically constrained by the proximity of gastrointestinal OARs, most notably the duodenum and stomach. Consequently, many patients receive doses in the intermediate-to-high range (BED_10_ <80 Gy). Whether further dose escalation beyond this range yields meaningful improvements in oncologic outcomes—or instead merely increases toxicity—remains controversial. A recent meta-analysis found no association between BED_10_ >70 Gy and 1-year LC rates (13). A recent comprehensive systematic review of prospective SBRT data further highlighted the variability in dose-fractionation regimens and the absence of consensus regarding the optimal BED threshold (14), underscoring the need for additional investigation.

This study contributes to this evolving body of evidence by evaluating the dose-survival relationship in a mono-institutional cohort of 46 patients treated with SBRT for pancreatic cancer. Unlike prior reports that largely focused on locally advanced (LA) disease, our cohort includes both LA and M1 patients, providing a broader real-world perspective. Importantly, this study does not aim to provide a definitive answer regarding the optimal BED threshold, but rather to add to the accumulating data informing future trial design and clinical decision-making. Specifically, we sought to determine whether BED_10_ ≥80 Gy provides superior clinical outcomes compared to the <80 Gy range, while also examining the influence of disease stage, chemotherapy, and other prognostic factors on this relationship.

## Materials and methods

### Study design and patient population

This retrospective study was approved by the Institutional Review Board of The First Hospital of Hebei Medical University. All patients provided written informed consent for SBRT treatment and for the use of their clinical data in research analyses at the time of treatment. Consecutive patients with histologically confirmed or clinically diagnosed pancreatic adenocarcinoma treated with SBRT between May 2021 and December 2023 were identified. Patients were classified as having locally advanced (LA) disease (AJCC Stage III), metastatic (M1) disease (Stage IV), or early-stage (Stage I/II) disease. Patients with recurrent disease following prior surgical resection were included but identified as a distinct subgroup for sensitivity analyses, given the potential for selection bias toward resistant disease biology.

All patients were discussed at a multidisciplinary pancreatic cancer conference and deemed unresectable or medically inoperable. Unresectability was primarily determined by vascular involvement (e.g., encasement of the superior mesenteric artery or celiac axis, or unreconstructable superior mesenteric/portal vein involvement) rather than tumor size alone, per standard oncologic criteria. Patients could have received prior chemotherapy, immunotherapy, or surgical resection. Salvage systemic therapy was administered at the discretion of the treating medical oncologist before and/or after SBRT.

### SBRT treatment planning and delivery

SBRT was delivered using the CyberKnife (Accuray, Sunnyvale, CA, USA) platform with the Synchrony Respiratory Tracking System for real-time motion management. Respiratory motion was managed through a combination of the Synchrony system (which correlates external respiratory motion with internal fiducial tracking) and, when indicated, abdominal compression, in accordance with AAPM Task Group 101 guidelines (15).

Patients underwent CT-guided implantation of 1–2 gold fiducial markers within or adjacent to the pancreatic tumor. The selection of fractionation was based on predetermined, protocol-defined criteria including: (1) tumor volume and location within the pancreas (head vs. body/tail), (2) proximity to OARs (duodenum, stomach, small bowel), and (3) patient performance status and comorbidities. Specifically, patients with tumors >3 cm in maximal diameter or those with tumors abutting the duodenal wall (>180°contact) were preferentially treated with a more fractionated regimen (5–6 fractions) to maintain OAR constraints. Dose was prescribed to an isodose line encompassing ≥95% of the planning target volume (PTV). The prescription isodose line was constrained between 68.3% and 73%, with a median prescribed isodose of 70% to achieve intratumoral dose heterogeneity.

Gross tumor volume (GTV) was defined as the radiographically visible tumor on simulation CT and diagnostic imaging. Clinical target volume (CTV) was equivalent to GTV. PTV was generated by adding a 3–5 mm margin to the GTV. Maximum point doses to the gastric wall, duodenal wall, and small bowel loops were maintained at or below the prescription dose. Dose constraints adhered to AAPM TG-101 guidelines (15).

BED_10_ was calculated as BED = nd[1 + d/(α/β)], where n is the number of fractions, d is the dose per fraction, and α/β = 10 for tumor effect. Patients were stratified into two groups based on received BED_10_: Group 1 received <80 Gy (delivered in 4–6 fractions); Group 2 received ≥80 Gy (also 4–6 fractions). The median BED_10_ was 72 Gy (range: 71.4–100 Gy); 31 patients received BED_10_ <80 Gy and 15 patients received ≥80 Gy.

### Follow-up and endpoints

The follow-up period concluded in October 2024, with a median follow-up of 12.0 months (range: 1–27 months) for the entire cohort. For the 33 deceased patients, the median follow-up was 12.0 months, while for the 13 surviving patients, the median follow-up was 18.0 months. Patients were followed with CT or PET/CT imaging at 3-month intervals for the first 2 years, then every 6 months thereafter. Local control was evaluated by comparing pre- and post-treatment imaging studies reviewed by the institutional radiology department as part of routine clinical practice: patients were classified as free from local progression if imaging reports indicated stable or decreased tumor size or FDG activity. Local failure was defined as radiographic tumor progression (increase in size or FDG activity). Of the 46 patients in the overall cohort, 19 patients lacked serial post-treatment imaging studies (CT or PET/CT) required for radiographic response assessment. These 19 patients were therefore excluded from PFS and LC analyses. The remaining 27 patients (20 in the <80 Gy group and 7 in the ≥80 Gy group) had at least one post-treatment imaging study and constituted the evaluable cohort for PFS and LC. All 27 evaluable patients experienced PFS events (i.e., radiographic progression or death), with no censored observations. Toxicity was graded per CTCAE v5.0. OS was calculated from the date of SBRT initiation. The primary endpoint was OS; secondary endpoints included LC, PFS, and treatment-related toxicity.

### Statistical analysis

Patient characteristics were summarized using medians and ranges for continuous variables and frequencies with percentages for categorical variables. Group comparisons were performed using the Mann–Whitney U test for continuous variables and Fisher exact test or chi-square test for categorical variables.

Survival analyses were performed using the Kaplan–Meier method with log-rank testing for group comparisons. OS was calculated for all 46 patients. PFS and LC were calculated only for the 27 patients with adequate imaging follow-up. For PFS analysis, all 27 evaluable patients experienced events (no censored observations). Multivariable Cox proportional hazards regression was performed to identify independent prognostic factors, including disease stage, chemotherapy, tumor size, BED_10_ dose group, and fiducial marker status. The proportional hazards assumption was verified using Schoenfeld residuals.

As an exploratory analysis, dose-response relationships were examined using logistic regression (1-year survival) and continuous Cox proportional hazards regression; detailed methods are provided in the **Supplementary Materials**.

Interaction testing was performed by including multiplicative interaction terms in separate Cox models: (1) locally advanced stage (LA, Stage III) × dose group (≥80 vs. <80 Gy), (2) metastatic stage (M1, Stage IV) × dose group, and (3) chemotherapy (yes vs. no) × dose group. The hazard ratio for each interaction term represents the ratio of hazard ratios across strata, and significance was evaluated using the Wald test.

Subgroup analyses were performed by fitting univariable Cox models within each stratum: disease stage (I/II, III/LA, IV/M1) and chemotherapy status. These analyses were exploratory and should be interpreted with caution given the small sample sizes within subgroups.

A sensitivity analysis was performed excluding 3 patients with recurrent disease who had received prior surgical resection followed by repeat CyberKnife treatment, to assess potential bias from their inclusion.

All statistical tests were two-sided, with alpha set at 0.05. Due to the exploratory nature of this analysis and the limited sample size, p-values should be interpreted as hypothesis-generating rather than confirmatory. No formal multiplicity adjustment was applied to secondary and exploratory analyses; therefore, all p-values from subgroup, interaction, and dose-response analyses should be interpreted as descriptive rather than confirmatory. Statistical analyses were performed using SPSS Version 27.0 and Python (lifelines, statsmodels, scipy).

## Results

### Patient and treatment characteristics

A total of 46 patients with pancreatic adenocarcinoma were included. The median age was 65.5 years (range: 41–84 years); 29 (63%) were male and 17 (37%) were female. ECOG performance status was 0 in 18 (39%), 1 in 24 (52%), and 2 in 4 (9%) patients. Tumor location was predominantly the pancreatic head (n=38, 83%). Disease stages included: I/II (n=10, 22%), locally advanced (III, n=17, 37%), and metastatic (IV, n=19, 41%). Twenty-four patients (52%) received chemotherapy, and 40 (87%) had gold fiducial markers implanted. Thirty-one patients received BED_10_ <80 Gy and 15 received ≥80 Gy. Of the 46 patients included in the OS analysis, 27 (58.7%) had adequate imaging follow-up for PFS and LC assessment, while 19 (41.3%) were excluded from these analyses due to the absence of serial post-treatment imaging. Among the 27 evaluable patients, 20 (74.1%) received BED_10_ <80 Gy and 7 (25.9%) received ≥80 Gy. Baseline characteristics were well balanced between dose groups (**Table 1**; all P > 0.05), though the largest non-significant differences were observed in disease stage distribution (Stage III: 45% vs. 20%; P = 0.265) and diabetes prevalence (23% vs. 40%; P = 0.299). The median follow-up time was 12.0 months for the entire cohort: 12.0 months for the 33 deceased patients and 18.0 months for the 13 surviving patients.

**Table 1:** Patient demographic and baseline clinical characteristics by dose group.

Characteristic	Total (N = 46)	BED_10_ <80 Gy (N = 31)	BED_10_ ≥80 Gy (N = 15)	P-value
Age, median (range)	65.5 (41-84)	65.0 (41-84)	67.0 (47-84)	0.597
Male, n (%)	29 (63)	20 (65)	9 (60)	1.000
Female, n (%)	17 (37)	11 (35)	6 (40)	
ECOG 0, n (%)	18 (39)	10 (32)	8 (53)	0.379
ECOG 1, n (%)	24 (52)	18 (58)	6 (40)	
ECOG 2, n (%)	4 (9)	3 (10)	1 (7)	
Stage I/II, n (%)	10 (22)	6 (19)	4 (27)	0.265
Stage III (LA), n (%)	17 (37)	14 (45)	3 (20)	
Stage IV (M1), n (%)	19 (41)	11 (35)	8 (53)	
Chemotherapy, n (%)	24 (52)	16 (52)	8 (53)	1.000
Recurrent disease, n (%)	3 (7)	3 (10)	0 (0)	0.541
Gold fiducial marker, n (%)	40 (87)	28 (90)	12 (80)	0.375
CA19–9 abnormal, n (%)	32 (70)	23 (74)	9 (60)	0.495
Diabetes, n (%)	13 (28)	7 (23)	6 (40)	0.299

ECOG, Eastern Cooperative Oncology Group; LA, locally advanced; M1, metastatic; BED_10_, biologically effective dose for α/β = 10, CA19-9, carbohydrate antigen 19-9.

### Survival outcome

**Figure 1:**
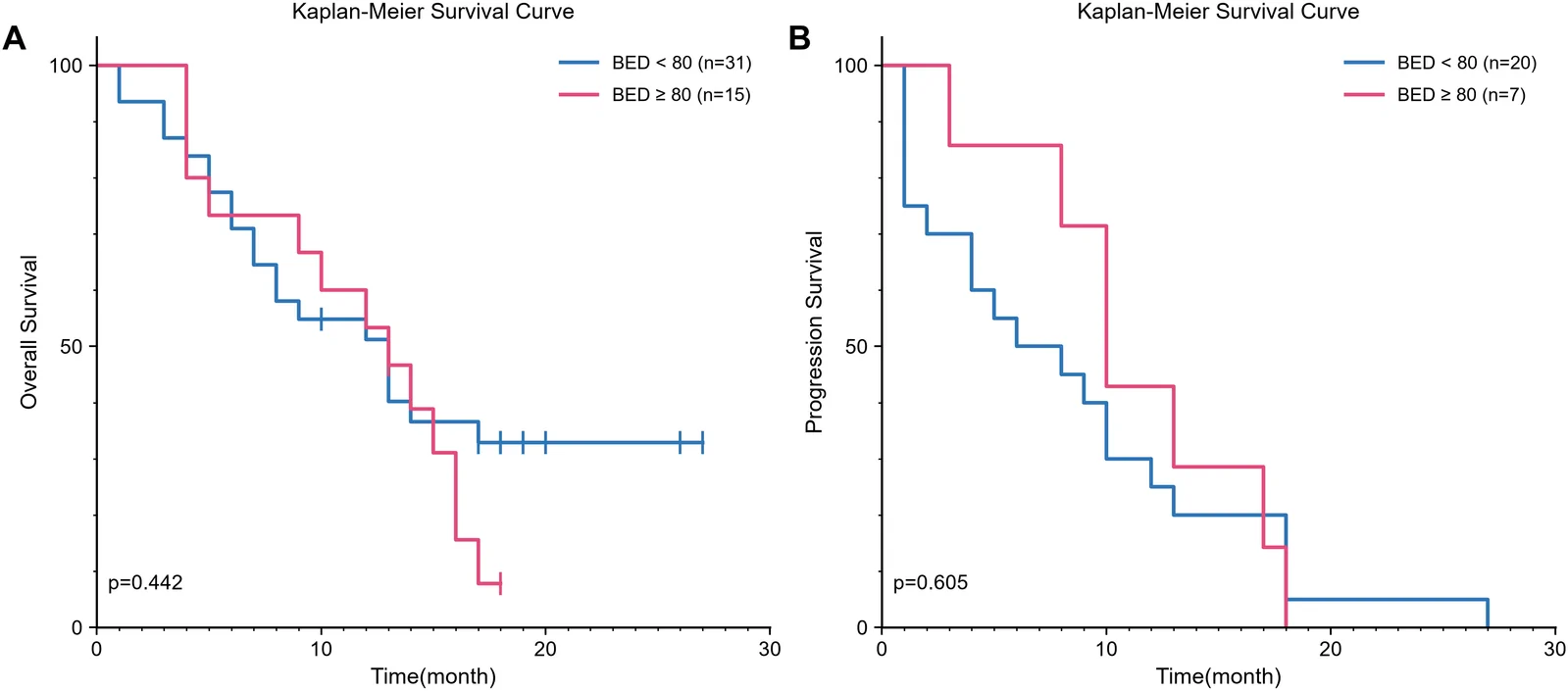
Kaplan–Meier plots for overall survival (A, p=0.442) and progression-free survival (B, p=0.605), measured from the date of treatment, comparing patients who were treated with BED_₁₀_ <80 Gy versus BED_₁₀_ ≥80 Gy. **(A)** Overall survival (OS) curves for all 46 patients, separated into BED_10_ < 80 Gy (n=31) and BED_10_ ≥ 80 Gy (n=15). Log-rank test showed no significant intergroup OS difference (p=0.442). Tick marks represent censored patients. **(B)** Progression-free survival (PFS) curves restricted to 27 patients with complete post-SBRT imaging follow-up, divided into BED_10_ < 80 Gy (n=20) and BED_10_ ≥ 80 Gy (n=7). Log-rank p=0.605 indicated no significant difference in PFS between low- and high-dose groups.

**Figure 2:**
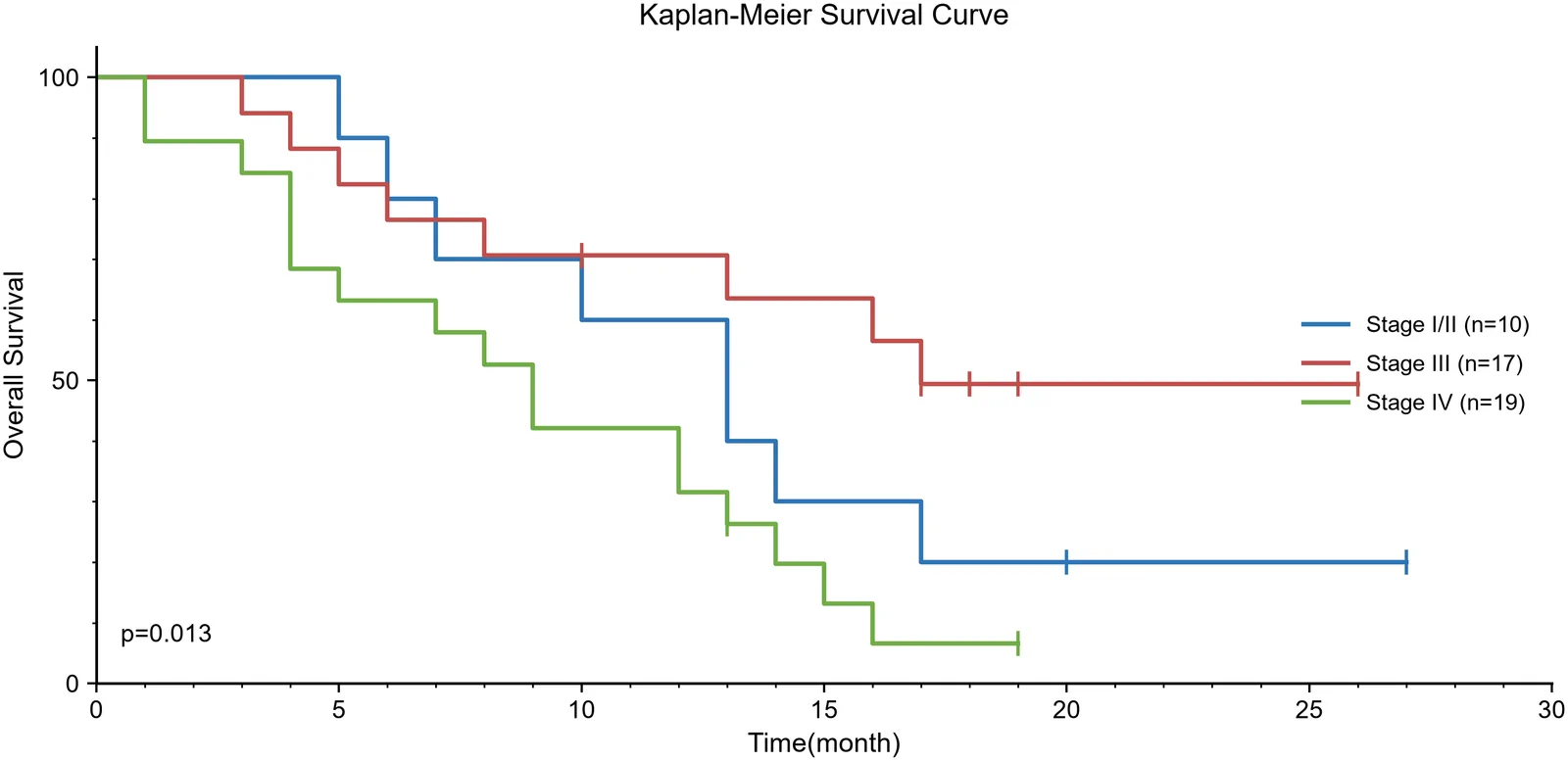
Kaplan–Meier overall survival (OS) stratified by disease stage. OS curves were plotted for stage I/II, locally advanced stage III (LA), and metastatic stage IV (M1) pancreatic cancer patients receiving SBRT. Log-rank test was used to compare survival differences across three groups (p=0.013).

The median OS for the entire cohort was 13.0 months (95% CI: 9.9–16.1). Kaplan–Meier survival curves stratified by dose group showed no significant difference (**Figure 1A**): the median OS was 13.0 months for <80 Gy and 13.0 months for ≥80 Gy. One-year OS rates were 51.2% (<80 Gy) and 53.3% (≥80 Gy), respectively (log-rank p=0.442). PFS was evaluated in the 27 patients with adequate imaging follow-up. All 27 patients experienced PFS events (radiographic progression or death), with no censored observations. PFS did not differ significantly between dose groups (**Figure 1B**): the median PFS was 8.0 months (95% CI: 5.6–10.4) for the <80 Gy group (n=20) and 10.0 months (95% CI: 6.0–14.0) for the ≥80 Gy group (n=7) (log-rank p=0.605). Notably, disease stage significantly impacted OS: LA patients had a median OS of 17.0 months versus 9.0 months for M1 patients (3-group log-rank p=0.013, **Figure 2**). Baseline characteristics of the 27 patients with evaluable imaging follow-up were comparable to those of the 19 patients without imaging follow-up across all assessed variables, including age, sex, ECOG performance status, disease stage, chemotherapy status, BED_10_ dose group, and fiducial marker status (all p > 0.05; **Supplementary Table 1**). Median OS did not differ between groups (13.0 vs. 12.0 months, log-rank p = 0.260), suggesting that the PFS and LC estimates derived from the evaluable subset are reasonably representative of the overall cohort.

On multivariable Cox regression (**Table 2**), BED_10_ dose group was not significantly associated with OS (HR 1.472, 95% CI: 0.663–3.265, p=0.342). Significant independent prognostic factors included metastatic disease stage (HR 0.274, 95% CI: 0.109–0.692, p=0.006, for non-metastatic vs. metastatic), absence of gold fiducial markers (HR 4.224, 95% CI: 1.512–11.801, p=0.006), larger tumor size (HR 0.399, 95% CI: 0.178–0.895, p=0.026, for <4 cm vs. ≥4 cm), and chemotherapy receipt (HR 0.410, 95% CI: 0.184–0.911, p=0.029, for chemotherapy vs. no chemotherapy). Among these, metastatic stage, marker absence, and larger tumor size were risk factors for poorer OS, while chemotherapy was associated with improved OS. Notably, chemotherapy was not significant in univariable analysis (HR 0.722, p=0.353) but emerged as an independent prognostic factor in the multivariable model, likely reflecting the preferential use of chemotherapy in patients with more advanced disease, which may have masked its true benefit in the unadjusted analysis. Given the limited sample size, this finding should be considered hypothesis-generating rather than indicative of a confirmable suppression effect. In univariable analysis, disease stage (HR 0.302, p=0.007), tumor size (HR 0.464, p=0.031), and gold fiducial markers (HR 3.705, p=0.005) were significant predictors of OS, while dose was not (HR 0.766, p=0.457). Complete univariable results are detailed in **Table 2**.

**Table 2:** Univariate and multivariate analysis of overall survival from treatment.

Factor	HR	(95% CI)	P-value
Univariate analysis			
gender	1.206	(0.583, 2.488)	0.613
age	0.839	(0.422, 1.668)	0.617
stage	0.302	(0.127, 0.717)	0.007 (P<0.05)
chemotherapy	0.722	(0.363, 1.436)	0.353
size	0.464	(0.231, 0.931)	0.031 (P<0.05)
location	1.780	(0.752, 4.211)	0.190
dose	0.766	(0.379, 1.547)	0.457
gold fiducials	3.705	(1.472, 9.323)	0.005 (P<0.05)
diabetes	1.311	(0.603, 2.853)	0.495
CA199	1.121	(0.533, 2.357)	0.763
Multivariate analysis			
stage	0.274	(0.109, 0.692)	0.006 (P<0.05)
chemotherapy	0.410	(0.184, 0.911)	0.029 (P<0.05)
size	0.399	(0.178, 0.895)	0.026 (P<0.05)
dose	1.472	(0.663, 3.265)	0.342
gold fiducials	4.224	(1.512, 11.801)	0.006 (P<0.05)

HR, hazard ratio; CI, confidence interval.

### Dose-response analysis

Dose-response analysis did not demonstrate a significant continuous relationship between BED_10_ and OS. Logistic regression yielded an odds ratio of 1.06 per Gy increase (95% CI: 0.97–1.16, p = 0.187), and continuous Cox regression yielded a hazard ratio of 1.01 per Gy (95% CI: 0.97–1.05, p = 0.749; **Supplementary Figure 1**). These results suggest that, within the studied BED_10_ range (71.4–100 Gy), a continuous gradient of dose effect on survival was not demonstrable. However, the narrow dose distribution within the <80 Gy group (26 of 31 patients received BED_10_ = 72 Gy) limits the ability to detect a continuous relationship within this subgroup.

### Interaction analysis

To examine whether the effect of dose on OS varied by patient subgroup, we performed interaction testing using Cox proportional hazards regression with multiplicative interaction terms (**Table 3**). No statistically significant interactions were detected: LA (Stage III) × dose (HR 0.77, 95% CI: 0.13–4.62, p = 0.773), M1 (Stage IV) × dose (HR 0.53, 95% CI: 0.13–2.15, p = 0.373), or chemotherapy × dose (HR 0.31, 95% CI: 0.07–1.27, p = 0.104). These analyses were *post-hoc* and exploratory, and the wide confidence intervals reflect limited statistical power for interaction detection in this relatively small cohort.

**Table 3:** Interaction analysis: effect of dose on OS across patient subgroups.

Interaction term	HR for interaction	95% CI	P-value
LA (Stage III) × dose	0.77	(0.13–4.62)	0.773
M1 (Stage IV) × dose	0.53	(0.13–2.15)	0.373
Chemotherapy × dose	0.31	(0.07–1.27)	0.104

LA, locally advanced; M1, metastatic; HR for interaction represents the ratio of HRs; p-values from Wald test.

### Subgroup analysis

Stratified analyses by disease stage and chemotherapy status (**Table 4**; **Supplementary Figure 2**) revealed no statistically significant dose effect within any subgroup: stage I/II (n = 10, HR 2.56, 95% CI: 0.57–11.57, p = 0.222), LA (n = 17, HR 1.08, 95% CI: 0.21–5.40, p = 0.928), M1 (n = 19, HR 0.76, 95% CI: 0.29–2.00, p = 0.574), with chemotherapy (n = 24, HR 0.75, 95% CI: 0.28–2.00, p = 0.564), and without chemotherapy (n = 22, HR 2.55, 95% CI: 0.88–7.36, p = 0.084). These subgroup analyses were exploratory, and the wide confidence intervals—reflecting small stratum sizes—preclude definitive conclusions about effect modification.

**Table 4:** Subgroup analysis: univariable cox models for dose effect within strata.

Subgroup	N	HR (BED_10_ ≥80 vs. <80)	95% CI	P-value
Stage I/II	10	2.56	(0.57–11.57)	0.222
LA (Stage III)	17	1.08	(0.21–5.40)	0.928
M1 (Stage IV)	19	0.76	(0.29–2.00)	0.574
With chemotherapy	24	0.75	(0.28–2.00)	0.564
Without chemotherapy	22	2.55	(0.88–7.36)	0.084

LA, locally advanced; M1, metastatic. HRs represent the effect of BED10 ≥80 Gy vs. <80 Gy within each subgroup.

### Local control

Local control was assessed in the 27 patients with evaluable imaging follow-up. The overall LC rate was 92.6% (25/27). Two local failures were documented, both occurring in the <80 Gy group; no local failures were observed in the ≥80 Gy group. Due to the limited number of LC events (n=2), formal comparative statistical analysis between dose groups was not feasible (Fisher exact test, p=1.0). These results suggest favorable local control with both dose ranges, though the small number of events precludes robust dose-group comparisons.

### Palliation of symptoms and toxicity

Prior to SBRT, 16 patients (34.8%) reported abdominal and/or back pain requiring medication. Following SBRT, this number decreased to 4 patients (8.7%), indicating effective symptom palliation. No grade 3 or higher acute gastrointestinal toxicity was observed in either dose group. Late toxicity data remain limited due to the short follow-up duration and high mortality rate.

### Sensitivity analysis

**Figure 3:**
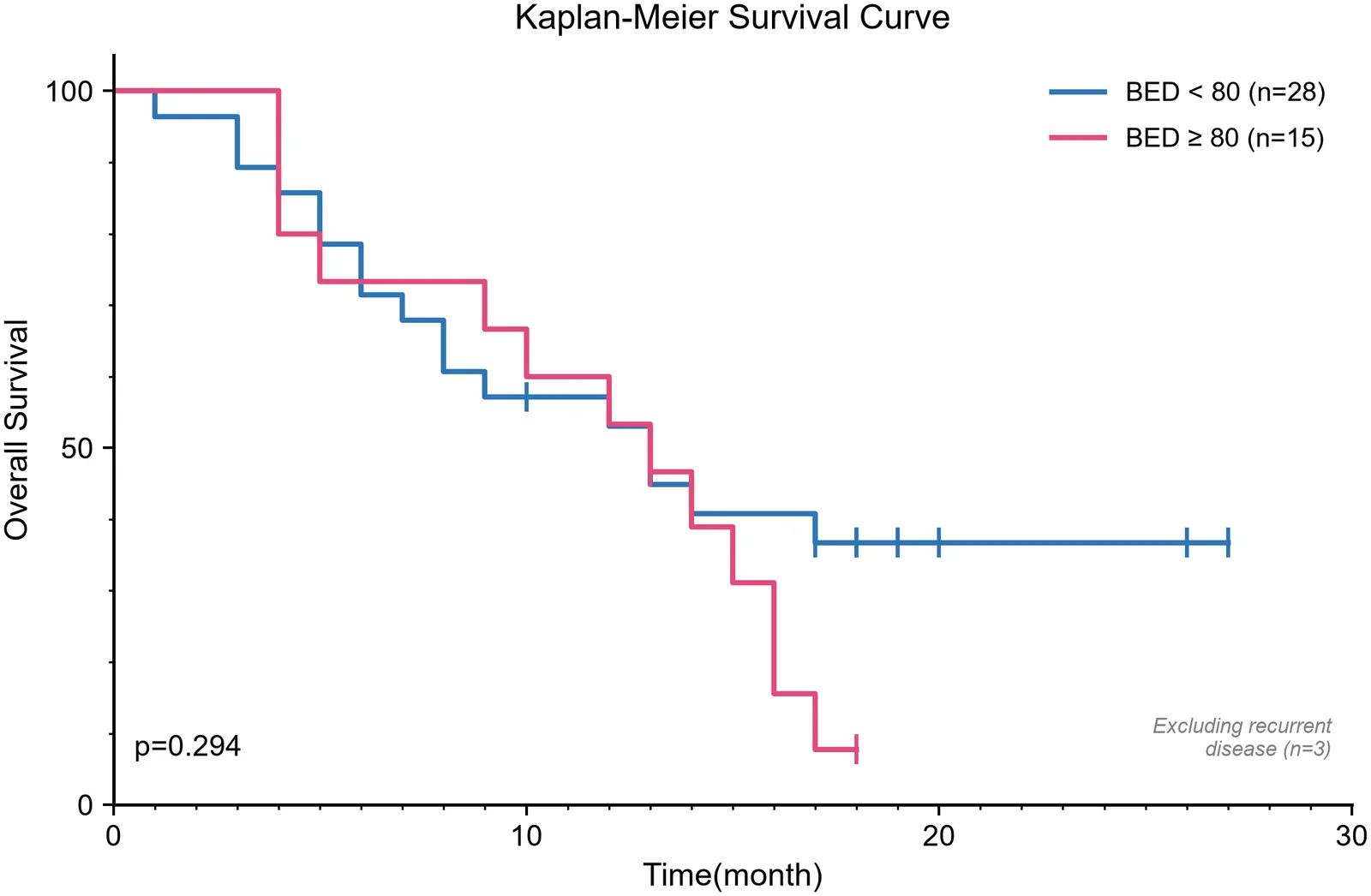
Sensitivity analysis of overall survival (OS) after excluding 3 patients with recurrent pancreatic cancer who received prior surgical resection. Kaplan–Meier curves compared OS between BED_10_ <80 Gy and ≥80 Gy groups. Log-rank p=0.294, median OS remained 13.0 months in both cohorts, consistent with primary survival results.

Excluding 3 patients with recurrent disease who had received prior surgical resection, the median OS remained 13.0 months for both <80 Gy and ≥80 Gy (28 vs. 15 patients; 17 vs. 13 events, log-rank p=0.294), consistent with the primary analysis (p=0.442). The 1-year OS rates were nearly identical between groups (53.1% vs. 53.3%), further confirming the absence of a dose effect on OS (**Figure 3**; **Table 5**).

**Table 5:** Sensitivity analysis: overall survival excluding recurrent resected cases.

Group	N	Events	Median OS (mo)	1-year OS rate
BED_10_ <80Gy	28	17	13.0	53.1%
BED_10_ ≥80Gy	15	13	13.0	53.3%

Log-rank p=0.294.

## Discussion

In this single-center retrospective analysis, we evaluated the relationship between SBRT dose and clinical outcomes in 46 patients with pancreatic cancer. Our principal findings are as follows: (1) no significant survival advantage was observed with BED_10_ ≥80 Gy compared to <80 Gy; (2) disease stage was the predominant determinant of OS; (3) an association was observed between fiducial marker absence and OS, though based on a limited number of patients; and (4) dose escalation was safe, with no grade ≥3 toxicity. These findings add to the accumulating evidence on dose-response in pancreatic SBRT and underscore the importance of patient selection and precise target localization in interpreting dose-outcome relationships. This analysis evaluates the BED_10_ <80 Gy versus ≥80 Gy threshold specifically, a comparison that, to our knowledge, has not been extensively examined in the existing pancreatic SBRT literature.

Our results align with the meta-analysis by Zaorsky et al. (13), which found no correlation between BED_10_ >70 Gy and 1-year LC rates across 19 pooled trials. However, they contrast with the MD Anderson experience reported by Krishnan et al. (9), where focal dose escalation (BED_10_ ≥70 Gy) was the only modifiable predictor of improved OS. Unlike many prior studies that focused exclusively on locally advanced disease, our cohort included a substantial proportion of patients with metastatic disease (41%), offering real-world SBRT outcomes in a population that is frequently excluded from dose-escalation studies but routinely encountered in clinical practice. Several factors may explain this discrepancy. First, the MD Anderson cohort was restricted to LA patients receiving induction chemotherapy, representing a more homogeneous and selected population. In contrast, our cohort included both LA and M1 patients, with the latter group dominated by systemic disease burden unlikely to benefit from local dose escalation. Second, the dose gradient in our study (71.4–72 Gy vs. 80–100 Gy) is narrower than the comparison in Krishnan et al. (<70 Gy vs. ≥70 Gy), which may attenuate detectable differences.

Based on 40 patients with fiducial markers versus 6 without, a notable finding of our multivariable analysis was that absence of gold fiducial markers was a strong independent predictor of poorer OS (HR 4.224, 95% CI: 1.512–11.801, p=0.006), with a hazard ratio exceeding that of all other prognostic factors including disease stage. This finding has particular relevance to CyberKnife-based SBRT, where the Synchrony Respiratory Tracking System relies on implanted fiducial markers for real-time internal tumor tracking. In the absence of fiducial markers, tumor localization must rely on external surrogate markers or skull-based tracking, potentially leading to larger PTV margins, increased dose to adjacent organs at risk, and compromised tumor dose coverage. Moningi et al. (16) retrospectively compared clinical outcomes between patients with and without fiducial markers in 96 pancreatic cancer patients treated with SBRT and found no significant difference in local recurrence (adjusted HR 0.6, p=0.59) or OS (adjusted HR 0.8, p=0.65) between groups. However, their cohort included patients treated on various SBRT platforms, and the critical dependence on fiducial markers for real-time tracking is unique to CyberKnife. Notably, review of the medical records revealed that all six patients without fiducial markers had declined the invasive placement procedure rather than being deemed technically ineligible. This argues against confounding by anatomical or technical factors as an explanation for the observed association. Nevertheless, this finding should be interpreted with considerable caution for several reasons. First, the small number of patients without markers (n = 6) results in a wide confidence interval (HR 4.224, 95% CI: 1.512–11.801) and limited statistical reliability; removal or reclassification of even one or two patients could substantially alter the estimate. Second, patient refusal of an invasive procedure may correlate with other unmeasured factors—such as health literacy, treatment adherence, or psychological characteristics—that could independently influence survival outcomes. Third, as a retrospective observation, this association does not establish causality. The finding is best considered hypothesis-generating and warrants prospective validation in larger cohorts that systematically capture reasons for non-placement before it can inform clinical decision-making.

Formal interaction testing did not reveal significant effect modification by disease stage or chemotherapy status, and subgroup analyses were similarly non-significant across all strata. However, these analyses were severely underpowered and should be considered hypothesis-generating. The absence of statistically significant dose effects within subgroups should be interpreted as absence of demonstrated effect in this cohort rather than evidence that dose escalation offers no clinical benefit in any subgroup. These interaction and continuous dose-response analyses were exploratory, and their primary contribution lies in demonstrating that the null dose effect was consistent across the examined subgroups and dose ranges rather than in providing definitive evidence of effect modification.

An important consideration in interpreting our findings is the evolution of SBRT technology. Our cohort was treated with CyberKnife using the Synchrony Respiratory Tracking System, which provides real-time motion adaptation. This contrasts with modern MR-guided adaptive SBRT approaches, such as those evaluated by Pavic et al. (17), where daily plan adaptation based on real-time MRI visualization of tumor and OARs may enable safer dose escalation. MR-guided SBRT with simultaneous integrated boost (SIB) strategies can selectively escalate dose to the tumor core while respecting OAR constraints, potentially achieving higher effective doses than conventional SBRT planning. The favorable local control in our series (92.6%) despite relatively modest BED_10_ values may partly reflect the precision of CyberKnife delivery with Synchrony tracking, though this technology does not provide the same adaptive capabilities as MR-guided approaches. Comparative studies of CyberKnife SBRT with MR-guided SIB approaches are warranted to determine whether improved treatment delivery translates to better outcomes.

The issue of tumor heterogeneity in radiation response merits discussion. Pancreatic cancer is characterized by a dense desmoplastic stroma that contributes to radioresistance (18). It is possible that the radio-biologically effective dose required to overcome this resistance exceeds the range achievable in our cohort (maximum BED_10_ = 100 Gy). Reyngold et al. (19) have advocated for truly ablative doses (BED_10_ >100 Gy) to maximize tumor control, and their prospective Phase 1 trial confirmed the safety of dose escalation up to 50 Gy in 3 fractions (BED_10_ = 133 Gy) (12). The favorable toxicity profile in our high-dose group suggests that further dose escalation may be feasible with appropriate patient selection and OAR sparing, particularly with advanced motion management and adaptive planning technologies.

We wish to explicitly acknowledge the following limitations of this study: (1) Small sample size (N = 46), with only 15 patients in the ≥80 Gy BED_10_ group, substantially limiting statistical power. (2) Heterogeneous patient population comprising both LA (Stage III, n=17) and M1 (Stage IV, n=19) disease, which complicates interpretation of OS. The significant prognostic impact of disease stage (HR 0.274, 95% CI: 0.109–0.692, p=0.006) underscores that metastatic disease biology may overwhelm any local treatment effect, and the inclusion of M1 patients dilutes the potential signal from local dose escalation in LA patients. We acknowledge that this heterogeneity limits the generalizability of our findings to homogeneous disease subgroups. (3) Retrospective, non-randomized design with potential selection bias in treatment allocation. Higher BED_10_ doses were preferentially delivered to patients with favorable OAR anatomy, which may confound the dose–outcome relationship. (4) The absence of a statistically significant dose–response relationship should be interpreted as an absence of demonstrated effect in this cohort rather than evidence that dose escalation beyond 80 Gy offers no clinical benefit. This distinction is critical given the limited statistical power and wide confidence intervals. (5) PFS and LC were evaluable in only 27 of 46 patients (58.7%), as 19 patients lacked serial post-treatment imaging due to loss to follow-up. This reduction in sample size limits statistical power for PFS and LC analyses. However, baseline characteristics and OS were comparable between patients with and without imaging follow-up (**Supplementary Table 1**), suggesting no systematic selection bias. (6) Single-institution experience, limiting external generalizability. (7) Limited systematic follow-up for late toxicity assessment due to the high mortality rate in this population, potentially underestimating late toxicity rates. (8) The narrow dose range within the <80 Gy group (26 of 31 patients received BED_10_ = 72 Gy) limits the ability to detect a continuous dose-response relationship within this subgroup. Furthermore, the resulting bimodal distribution of BED_10_—with a gap of nearly 10 Gy between the two dose clusters and minimal overlap—further limits the interpretability of the continuous dose-response analysis, which assumes a graded exposure across the full dose spectrum. This may partially explain the absence of a significant dose effect. (9) An additional limitation concerns the imaging-based response assessment in this study. Applying RECIST 1.1 criteria to evaluate tumor response after SBRT has inherent limitations: relying solely on CT-measured lesion size in the post-radiotherapy setting is fundamentally flawed, as radiation-induced fibrosis, necrosis, and inflammatory changes can mimic tumor progression on CT imaging, making assessment based solely on lesion dimensions unreliable. (10) The multivariable Cox model included five covariates with approximately 34 events, yielding approximately 6.8 events per variable (EPV). This is below the commonly cited threshold of 10–15 EPV, and the model estimates should be interpreted with appropriate caution regarding potential overfitting and instability (20, 21).

## Conclusion

In this single-center retrospective analysis, SBRT for pancreatic cancer achieved favorable local control with an acceptable safety profile. However, BED_10_ ≥80 Gy was not associated with a statistically significant survival benefit compared with <80 Gy. Independent prognostic factors included disease stage, tumor size, and chemotherapy. An exploratory association between fiducial marker absence and OS warrants prospective validation. Given the limited sample size and heterogeneous patient population, these findings should be interpreted as hypothesis-generating. Prospective randomized trials with homogeneous cohorts are needed to establish the optimal dose–fractionation regimen for pancreatic SBRT.

## Data Availability

The raw data supporting the conclusions of this article will be made available by the authors, without undue reservation.

## Supplementary material

Supplementary Figure 1 Continuous dose-response analyses of BED_10_ for overall survival (OS). **(A)** Logistic regression model estimating the probability of 1-year survival per 1 Gy increment of BED_10_ (OR = 1.06, p=0.187). **(B)** Continuous Cox proportional hazards regression evaluating instantaneous mortality risk per 1 Gy increase in BED_10_ (HR = 1.01, p=0.749). Grey shaded areas indicate 95% confidence intervals of fitted effect estimates.

Supplementary Figure 2 Forest plot of subgroup Cox regression analyses evaluating the impact of high BED_10_ (≥80 Gy versus <80 Gy) on overall survival (OS).Subgroup stratifications included disease stage (stage I/II, locally advanced stage III [LA], metastatic stage IV [M1]) and chemotherapy administration status (with vs. without concurrent chemotherapy). The horizontal lines represent 95% confidence intervals (CIs) of hazard ratios (HR), and solid squares denote point estimates of HR for each subgroup. The vertical reference line at HR = 1 indicates no survival difference between the two dose groups. All subgroup comparisons yielded p-values > 0.05, demonstrating no statistically significant survival benefit associated with escalated ablative BED_10_ in any stratified population.
