# Supplementary material for: Biologically effective doses for α/β = 10 (BED10) of <80 Gy versus ≥80 Gy in stereotactic body radiotherapy for pancreatic cancer: a retrospective single-center analysis
Source: Front Oncol. 2026 Aug 26;16:1850091. doi: 10.3389/fonc.2026.1850091 (PMC13553395; doi:10.3389/fonc.2026.1850091)
Supplement: Supplementary file 1 [file Table1.docx]

**Supplementary Table S1. Baseline characteristics of patients with versus without evaluable imaging follow-up.**

| **Characteristic** | **With Imaging (N=27)** | **Without Imaging (N=19)** | **p-value** |
| --- | --- | --- | --- |
| Age, median (range) | 65 (41-84) | 67 (47-84) | 0.326 |
| Male, n(%) | 17 (63) | 12 (63) | 1.000 |
| Female, n(%) | 10 (37) | 7 (37) |  |
| ECOG 0, n(%) | 9 (33) | 9 (47.5) | 0.565 |
| ECOG 1, n(%) | 15 (56) | 9 (47.5) |  |
| ECOG 2, n(%) | 3 (11) | 1 (5) |  |
| Stage I/II, n(%) | 4 (15) | 6 (31.6) | 0.582 |
| Stage III (LA), n(%) | 11 (41) | 6 (31.6) |  |
| Stage IV (M1), n(%) | 12 (44) | 7 (36.8) |  |
| Chemotherapy, n(%) | 16 (59) | 8 (42) | 0.370 |
| BED_10_ <80 Gy, n (%) | 20 (74) | 11 (58) | 0.341 |
| BED_10_ ≥80 Gy, n (%) | 7 (26) | 8 (42) |  |
| Gold fiducial marker, n(%) | 23 (85) | 17 (89) | 1.000 |
| CA19-9 abnormal, n(%) | 20 (74) | 12 (63) | 0.522 |
| Diabetes, n(%) | 9 (33) | 4 (21) | 0.510 |
| OS, months, KM median | 13.0 | 12.0 | 0.260 |

*Abbreviations: ECOG, Eastern Cooperative Oncology Group; Abbreviations: LA, locally advanced; M1, metastatic; BED_10_, biologically effective dose for α/β = 10, CA19-9, carbohydrate antigen 19-9, OS, overall survival; KM, Kaplan-Meier.*
